# Exploring primary care level provider interpretation and management of potential breast and cervical cancer signs and symptoms in South Africa

**DOI:** 10.3332/ecancer.2021.1298

**Published:** 2021-09-30

**Authors:** Jennifer Moodley, Jane Harries, Suzanne Emilie Scott, Amos Deogratius Mwaka, Smiji Saji, Fiona Mary Walter

**Affiliations:** 1 Women’s Health Research Unit, School of Public Health and Family Medicine, Faculty of Health Sciences, University of Cape Town, Observatory, Cape Town 7925, South Africa; 2 Cancer Research Initiative, Faculty of Health Sciences, University of Cape Town, Observatory, Cape Town 7925, South Africa; 3 South African Medical Research Council Gynaecology Cancer Research Centre, Faculty of Health Sciences, University of Cape Town, Observatory, Cape Town 7925, South Africa; 4 Centre for Oral, Clinical and Translational Sciences, Faculty of Dentistry, Oral and Craniofacial Sciences, King’s College London, London SE1 9RT, UK; 5 Department of Medicine, School of Medicine, College of Health Sciences, Makerere University, Upper Mulago Jill Road, PO Box 7072, Kampala 256, Uganda; 6 The Primary Care Unit, Department of Public Health and Primary Care, University of Cambridge, Cambridge CB1 8RN, UK; 7 Wolfson Institute of Population Health, Barts and The London School of Medicine and Dentistry, Queen Mary University of London, London E1 4NS, UK; a https://orcid.org/0000-0002-9398-5202; b https://orcid.org/0000-0001-7359-8419; c https://orcid.org/0000-0001-5536-9612; d https://orcid.org/0000-0001-7952-2327; e https://orcid.org/0000-0003-0002-6326; f https://orcid.org/0000-0002-7191-6476

**Keywords:** primary health care providers, cancer symptom interpretation

## Abstract

**Objectives:**

Women with potential breast and cervical cancer symptoms in South Africa (SA) usually self-present to primary health care (PHC) clinics. The aim of this study was to explore PHC provider interpretation and management of potential breast and cervical cancer signs and symptoms.

**Methods:**

In-depth interviews with PHC providers incorporating vignettes were conducted between April and May 2019 in two sites in SA. Four vignettes (two breast and two cervical) were developed by the research team to capture aspects of provider symptom interpretation, reasoning, actions and challenges. The content of the vignettes was informed by a preceding community-based survey and qualitative interviews with symptomatic women. Interviews were audio recorded, transcribed verbatim and analysed using a thematic analysis approach.

**Results:**

Twenty-four PHC providers were interviewed (12 urban, 12 rural; median age: 43 years). Four main themes relating to clinical assessment and reasoning; referral and feedback challenges; awareness of breast and cervical cancer policy guidelines and training and education needs emerged. Vignette-prompted questions relating to presenting symptoms, and possible accompanying symptoms and signs, demonstrated comprehensive proposed history taking and clinical assessment by PHC providers. Cancer was considered as a potential diagnosis by the majority of PHC providers. PHC providers also considered the possibility of infectious causes for both breast and cervical vignettes indicating they would ask questions around human immunodeficiency virus status, use of anti-retroviral therapy, and, for those with cervical symptoms, would need to rule out a sexually transmitted infection. Sexual assault was considered in assessing the cervical symptom scenarios. Providers raised issues around cumbersome booking systems and lack of feedback from referral centres. The need for provider and patient education and training to improve timely diagnosis of breast and cervical cancer was raised. Most providers were not aware of current breast or cervical cancer policy guidelines.

**Conclusion:**

Clinical assessment at PHC level is complex and influenced by local health issues. Providing context-relevant training and support for PHC providers, and improving referral and feedback systems, could assist timely diagnosis of women with symptomatic breast and cervical cancer.

## Introduction

Breast and cervical cancer are leading causes of cancer morbidity and mortality in South Africa (SA), with breast cancer being the most commonly diagnosed cancer and cervical cancer the leading cause of cancer deaths among women [1, 2]. Both cancers have been recognised as priority diseases by the South African National Department of Health, with recent policy guidelines emphasising the importance of early diagnosis of symptomatic disease as it enables more opportunities for curative treatment and improved outcomes [3, 4]. This emphasis resonates with the World Health Organization’s recent positioning statement that recognition of symptomatic cancer at an early stage is one of the most effective public health measures in cancer control [5].

SA has a tiered public sector health care system (primary, secondary and tertiary levels). Typically, women with potential breast and cervical cancer symptoms will self-present to primary health care (PHC) clinics and will be initially assessed by a PHC provider. The majority (80%) of first contact care and assessment is by PHC nurse providers [6]. PHC providers attend to patients with a range of symptoms and undifferentiated health problems on a daily basis and need to prioritise among the many potential differential diagnoses. Whilst potential cancer symptoms are common, cancer is relatively rare. Research in high-income countries has shown that the vast majority of patients that present with possible cancer symptoms do not end up with a cancer diagnosis [7–11]. Local studies have shown that the presence of co-morbidities can complicate referral and cancer diagnosis [12, 13]. In one South African study, women with co-morbidities had a significantly longer diagnostic interval for breast cancer compared to those without co-morbidities [13]. Studies conducted elsewhere in Africa have shown that PHC providers have limited breast and cervical cancer knowledge. A study in Nigeria showed that 58% of PHC providers had fair or poor breast cancer knowledge [14]. Another Nigerian study reported that the majority of PHC workers interviewed showed poor understanding of cervical cancer disease, symptoms and risk factors [15]. In a needs assessment conducted in Rwanda, 60% of general practitioners based at district level hospitals identified a need for more information about various areas of cancer management, including diagnostic skills to detect cancers earlier [16]. PHC providers have a challenging but pivotal role in appraising women’s symptoms, assessing the likelihood of the symptoms being due to cancer and deciding whether to refer patients to secondary level facilities for further diagnostic tests and assessments. A timely and accurate initial appraisal of potential symptoms at the PHC level is a critical step in the cancer diagnostic pathway, yet little is known about factors influencing this assessment in low- and middle-income settings, including in SA. This qualitative sub-study formed part of a larger study aimed at better understanding pathways to care for women with possible breast and cervical cancer symptoms. We used both quantitative and qualitative methods to meet the overall study aim [17–20].

In this sub-study, we aimed to provide an in-depth, more nuanced understanding of PHC providers experience and clinical practices interpreting and managing potential breast and cervical cancer signs and symptoms.

## Methods

### Study design, population and setting

Qualitative research was deemed important and suitable to our research objectives. We used a qualitative in-depth interview (IDI) study design augmented by vignettes. The study was conducted between April and May 2019 at one urban and one rural site in SA. Details on the study site have been described previously [20, 21]. The research co-ordinator approached health facility managers in both research sites, explained the overall purpose of the study and requested a list of PHC providers who would encounter women with symptoms in their daily practice. We obtained information on professional status, level of experience, age and gender so that we could purposively recruit a mix of participants in both rural and urban sites. We then approached participants, invited them to participate in the study and set up a suitable time to be interviewed. Written informed consent was obtained from providers prior to the interview process. Consent (written) was also obtained to record all interviews. Confidentiality and anonymity were ensured, and participants were assured that responses would not affect their employment in any way.

### Data collection

Interviews were conducted by female research assistants trained in qualitative research methods, in a private room in the health care facility and were 40–60 minutes duration. All interviews were audio recorded and conducted in isiXhosa or English. Interviews were transcribed verbatim and translated into English by an independent transcriber.

Four vignettes were developed by the research team: two on breast symptoms and two on cervical symptoms. Vignettes are brief stories or scenarios that describe hypothetical situations to which a participant responds [22, 23]. Our vignettes were developed to capture aspects of provider symptom interpretation; reasoning, judgements and actions related to investigations and management; referral and feedback processes and consideration of infective or alternative diagnoses. The content of the vignettes was informed by findings from the preceding community-based cross-sectional survey, qualitative IDIs with symptomatic women and from drawing on the literature [18, 20]. Briefly, the vignettes described a 50-year-old woman with diabetes mellitus and a breast lump (scenario 1); a 50-year-old woman with diabetes mellitus and breast skin changes (scenario 2); a 35-year-old woman with human immunodeficiency virus (HIV), a vaginal discharge and post coital bleeding (scenario 3) and a 60-year-old woman with post-menopausal vaginal bleeding (scenario 4) (Appendix A). The interview guide and vignettes were piloted, and changes were made to improve flow and clarity of the vignettes. The vignettes were presented to participants in stages, with prompts to explore provider opinions and reasoning. Each provider was presented with two scenarios (one breast and one cervical) and we ensured an equal distribution of the two scenarios between both rural and urban sites. Study participants were all presented the scenarios in the same order – one breast and one cervical scenario. At the end of the scenario, providers were also asked about policy and guidelines related to the management of possible breast and cervical cancer in SA.

Interviewers recorded reflective fieldnotes after each interview which provided added context when reviewing the transcripts. Socio-demographic information was collected prior to the interview and included age, gender, professional status and years of experience including number of years working at the health care facility.

### Data analysis

We adapted aspects of the framework method to enhance thematic analysis by following the different stages of transcription, familiarisation with the interview, coding, developing and applying an analytical framework, charting data into the framework matrix and interpreting the data [24]. Initial categories for analysing data were drawn from the interview guide and vignettes, and then themes and patterns were identified after reviewing the data. The transcripts were coded by JH using the qualitative software package NVivo 12 Pro which facilitated the sorting and management of the data. SS sorted and coded the clinical data, capturing the key elements of responses related to history taking, clinical examination and investigations in a matrix framework (see Appendix B). The clinical coding was reviewed by JM and FMW. A code book with 30 codes, 19 *a priori* codes and 11 emergent codes was developed by JH based on the research objectives and interview guide and then shared with the research team and cross checked for coder variation. Coding discrepancies were resolved through discussion and consensus. Memos were recorded alongside the coding process and were useful in exploring relationships of links across categories, or reflections about a particular phenomenon. The data were then reviewed for major trends, crosscutting themes and issues for further exploration. Given the common themes occurring across the interviews, we concluded that data saturation had been achieved as little new information related to the main research questions emerged from the data.

To address dependability, we established an audit trail by developing a detailed track record of the data collection process, coding was undertaken by more than one person and coding was checked amongst the study team for coder variation. Coding discrepancies were resolved through discussion and consensus. For confirmability, all interviewers kept a reflective journal and we held regular investigator and researcher team meetings to discuss research progress. Further, data collection used a range of different interviewing techniques, vignettes and more structured questions with study respondents. Transferability was addressed through purposeful sampling to represent a range of respondents in both urban and rural sites. The findings of the study have been reported following the Consolidated Criteria for Reporting Qualitative Research [25].

### Ethical considerations

Ethical approval was obtained from the Human Research Ethics Committee, University of Cape Town (859/2017). Permission was also obtained from the local health departments to undertake research in the health care facilities. All study participants provided written consent to be interviewed and recorded.

## Results

A total of 24 PHC providers were interviewed (12 urban and 12 rural). The participant median age was 43 years (inter-quartile range: 27–59 years). The majority were female (20/24). Fifteen of the participants were professional nurses, five clinical nurse practitioners, one community service nurse and three were operations managers. Results are presented according to the main themes which included clinical assessment and reasoning; referral and feedback challenges; awareness of breast and cervical cancer policy guidelines and training and education needs.

### Clinical assessment and reasoning

#### Assessments for cancer

PHC provider accounts of the history that would be taken were comprehensive, with detailed questions around the presenting symptoms and on possible accompanying symptoms linked to the vignette scenario (see Appendix B). Similarly, descriptions of clinical examinations were detailed, related to the presenting symptom and included a full clinical breast examination (scenarios 1 and 2), vaginal examination with a speculum (scenarios 3 and 4) and for both a more general ‘head-to-toe’ assessment.

***For breast symptoms***, history taking included questions around physical symptoms such as pain, size and texture of lump, and duration of symptoms. In addition to physical symptoms, providers also asked about familial history of breast cancer. Providers suggested similar symptom history taking questions for both breast symptom scenarios.

*The first question is I’m going to ask the woman, when did she first notice the lump, is the lump growing or not growing; is the lump painful or not painful… And I am going to ask her if she is taking her medication well and ask her about the history of family and cancer – any cancer in the family* (Scenario 1 rural)

Providers also mentioned the importance of asking the patient about nipple and breast skin changes and similarly noted any changes during their clinical examination. A provider noted that skin changes, especially colour and skin texture and appearance (peau d’ orange) could be indicative of breast cancer. Checking for lymph nodes under the axilla was also deemed important to rule out breast cancer.

*You also ask about the changes. As you already told me there is changes on the breast, but you check if the change is in the nipple or on the skin. you also ask the client, is there any lump on the breast …So we talk about the lymph nodes, is there any lymph nodes on the armpit. So those are the things we first want to know and you check all those before you do your examinations... you can check those changes because if you query cancer there can be a change on the skin or sometimes it becomes orange or any discolouration on the skin or on the nipple*.…(Scenario 2 urban)

Some providers suggested total body examinations from ‘head to toe’ for breast changes, possibly to rule out other underlying conditions (anaemia, jaundice cyanosis, scoliosis).

*Examine the client from head to toe. She must take off all the clothes so that we can look and examine her… for the nurse to check the head and the eyes and the mouth, all the parts around the head because as a nurse you’re going to look for other problems except the one she comes with like maybe she’s got a problem with anaemia …you check if this rash is septic or is just a rash or is reddish and you just do the massage on the breast… to see if maybe there’s no lumps around the breast ..maybe this rash coming from inside the breast* – (Scenario 2 rural)

***For cervical symptoms***, the scenarios were often linked to other possible causes of vaginal bleeding such as hormonal contraception, possible miscarriage or pregnancy, retained products of conception post abortion and possible sexually transmitted infection (STI) and sexual violence (see later **Ruling out other infectious diseases** and **Querying sexual violence**).

*It is important because we want to check if this vaginal discharge is caused by a sexually transmitted infection, or is it thrush which is not sexually transmitted, it can be any kind of discharge so that’s what we want to exclude by asking that question with the symptoms that she’s presenting with it can be either between the sexually transmitted infections and signs of cancer, miscarriage or even after an abortion, then maybe there was products of conception that were left inside the uterus, So that can give her those signs that she’s presenting with, so asking those questions help me decide, this patient could likely be suffering from this or that* (Scenario 3 urban)

Almost all providers indicated that they would perform a Pap smear to rule out cervical cancer (for Scenario 4) as post-menopausal bleeding could be indicative of cervical cancer. However, some noted that they would not do a Pap smear if there was too much bleeding with a large lesion (as per scenario).

*You ask when the bleeding started because this client is not supposed to bleed again, that is the first thing… you suspect as the nurse that there is something wrong ... And you are not even going to do anything, you are not going to do a vaginal examination or anything, that is not what you are going to do because you suspect that there is cervical cancer … menopause long ago there’s nothing you’re going to do, you are going to refer this person to the doctor immediately; because you can’t do a smear on a bleeding client* (Scenario 4 rural)

Some providers recounted how they would examine the patient from ‘head to toe’ looking for generalised signs of bleeding, followed by a vaginal examination including for the presence of genital warts.

*First of all you need to do a physical examination on the whole body … I want to check if everything is normal from head to toe, to check if she’s not pale, there are no sores on the body and no rash before I do the vaginal examination* (Scenario 3 rural)

#### Considering HIV

Questions around HIV status and use of anti-retroviral therapy (ART) were an important part of history taking and influenced clinical reasoning and guidance.

*She is HIV positive. First of all I am going to ask if she is taking her treatment [ART] well. I am going to ask the client if she is using a condom and concerning her complaint I am going to ask all that, how many partners she has, when the bleeding started and then I am going to ask if she’s had a Pap smear before and then if the answer is no I’m going to continue with counselling that every woman that is on ART should do a Pap smear yearly* (Scenario 3 rural)Some providers (both rural and urban) noted that an HIV status could further compound the risk of cervical cancer**.**

*She’s HIV positive, the risk is much higher now for that cervical cancer as well, so that is why we need, to be much more vigilant with her* (Scenario 3 urban)

…*if a client is HIV positive you should do a Pap smear from 30 years of age, ….We want to exclude cervical cancer* (Scenario 3 rural)

For women with breast symptoms, a history of use of HIV medication (efavirenz) was considered important as it is a possible cause of breast lipodystrophy.

*The medication can help you to diagnose this client … For example, I’m working on ART, so if the client maybe comes with an enlarged breast, so that is where I know this client is on efavirenz and complaining about the enlargement of both breasts. So, it can be caused because of the efavirenz. So that is why it is very important to also check what kind of medication the client is on* (Scenario 2 urban)

#### Ruling out other infectious diseases

For the scenarios related to possible cervical cancer symptoms, ruling out a STI was deemed important. A provider described how she would establish whether symptoms (vaginal discharge, bleeding) could be attributed to an STI due to unprotected sex or retained products of conception, versus a non-infective condition such as thrush or a miscarriage or whether signs of possible cervical cancer.

A smelly, yellow vaginal discharge in an HIV positive woman was viewed by some providers as an indicator of an STI.

*Like I said the yellow discharge, obviously that’s clear that it’s an STI, you will ask if it’s smelling, then you know how to treat what type of infection it* is (Scenario 3 urban)*When was she diagnosed with HIV, has she been taking treatment or not, … How many sexual partners does she have, is she using a condom and when did she notice this unusual discharge and what else is there, does she have low abdominal pains, itchiness or any other problems and for how long has she been having this discharge and when did the bleeding start?* (Scenario 3 urban)

In relation to breast skin and colour changes, a provider noted that skin changes might be due to cellulitis, a breast abscess or mastitis and needed to be ruled out prior to assuming symptoms might be attributed to breast cancer.

*And then, I think also the colour of the lump or the size of the lump, I think is also important because also defining it as to what might be the conditions. Because there might be cellulitis, there may be breast cancer, all those things, they may manifest in the same manner you see…, we need to establish as to whether or not she has skin problems generally or not the skin problem can also affect the breast like breast abscess or mastitis* (Scenario 1 rural)

#### Querying sexual violence

Rape or sexual assault was considered a possible causative factor for the vaginal bleeding symptom described in Scenario 4 but did not arise in the history taking for Scenario 3. Often this questioning was related to the high levels of crime in SA.

*The first question I will ask, because our country has no law; has she been raped…*

*Is there any trauma, how long and when did this bleeding start, because of her age she shouldn’t bleed*…. *Because all these old mothers are being raped nowadays because there is no law here in South Africa. Maybe someone broke into her house at night and then raped her and that caused the bleeding or maybe the trauma by being abused by the thugs or robbers in her house* (Scenario 4 urban)

*Is there any history of assault? Because maybe if in cases of assault maybe, bleeding can occur in cases of rape* (Scenario 4 rural)

### Referral and feedback challenges

Providers raised a range of health systems issues in the process of diagnosis and treatment of women with potential breast or cervical cancer symptoms. These ranged from cumbersome booking systems to the way test results were received, tracked and followed up.

Most providers indicated that doctors played a key role in terms of patient management and referral. In situations where there were no facility, doctors available at primary care clinics providers would refer directly to a higher-level facility by making a telephonic appointment and/or providing the patient with a referral letter to take to the referral centre.

*The referral process in all the facilities is that it has to go via the doctor before they can accept a client it has to go via a doctor in the primary health care facility first and then to a tertiary hospital* (Scenario 1 urban)

Depending on where the health care facility was located, investigation or test results were received in different ways. In rural facilities, results were received via a courier, whereas in urban facilities with better infrastructure, providers were able to view results online. Informing patients of their test results was cumbersome and inefficient for both patients and providers. Whilst providers were able to eventually receive results, the system of contacting patients was complicated, relying on telephone contact, using community workers to access patients, or relying on patients returning to receive their results or, in some cases, relying on the referral doctor.

A provider explained how lines of communication and feedback were dependent on an *ad hoc* system and not a formal feedback process:

*The only thing is the curiosity from the nurse to keep on asking the doctor, but sometimes we always get the same answer, ‘No, I’m still waiting, that patient is still in hospital,’ you know, yes that is the problem. The only solution that can help us is for us to have direct referral system of these cases* (Scenario 2 urban)

Providers also voiced concerns related to lack of feedback, often relying on community workers to follow-up and in some instances finding out from others that a patient had died:

*As our nurses here we need a back-referral system and this was discussed for a long time about the referral system; but nothing is happening, and you have to know what happened to the client that you referred, and not hear from other people that the patient has passed away… You need to know how the doctor managed the patient, we have an interest in that but it’s not happening. I call, we phone, to give results, and if she doesn’t have the phone, we have the community workers they do the follow up* (Scenario 4 rural)

*We want to know what is happening with the client that we referred. If the referral system could improve – the back-referral system,… As I said, the back-referral system is bad, they don’t come back to us and tell us what was done on the client, there’s no back-referral system.[feedback]* (Scenario 1 rural)

*Interviewer : is the nurse in this situation likely to get feedback from the referral centres? Respondent: only for the colposcopy, that’s when you are able to get feedback. But for the other cases, unless you are lucky enough to, for the patient to come back and tell you what happened* (Scenario 4 urban)

### Awareness of breast and cervical cancer policy guidelines

Most providers interviewed were not aware of the existing breast or cervical cancer policy guidelines or their content nor had the opportunity to review the policies. A few providers mentioned that policy and guidelines might be available at their facility.

*‘no ….I can’t remember now … I don’t have the guidelines with me, but they are in a file, but I know what you are supposed to do. . . . I don’t want to lie, … I don’t remember’,* (Scenarios 1 and 3 urban)

*Because this* [breast cancer*] is a well-known condition, so I believe that there are policies for it but myself I’ve never come across those policies* (Scenario 2 rural)

For those few providers who had some knowledge of the cervical cancer screening policy, it was mostly related to screening guidelines (age and time intervals) including for HIV positive women; however, these providers were also unsure of where to locate the guidelines in their facility should they need to refer to it.

*There are guidelines because we do, this Pap smear that we do at 30 – Pap smear, 40 – Pap smear, 50 – pap smear, those are the guidelines…3 years every 3 years if HIV positive* (Scenario 3 urban)

Some providers whilst being familiar with the SA cervical and breast cancer guidelines did express an interest in engaging with the policy and guidelines.

*A provider located in the rural site requested information on current cancer policies to increase her knowledge about how to manage the types of scenarios described* (reflections rural)

### Training and education needs

Providers raised the need for education and training to improve timely access, diagnosis and treatment spontaneously. Regular educational workshops on breast cancer detection and diagnosis were deemed important to improve timely diagnosis and treatment of breast cancer.

*Yes, for me I think there should be more workshops for us Clinical Nurses to attend because most of the people if you can see the stats they are dying because of breast cancer, meaning which we are misdiagnosing here in the clinics. If we can be sure of what we are doing when we assess cancer and we assess the patients here first, we can detect it at an early stage because hospitals are full of breast cancer patients. And you ask yourself how and when the clinics missed these people; because when they reach us it’s too late, we should detect then when it starts here at the clinic. If we can have regular workshops, even twice a year, that’s fine* (Scenario 2 urban)

Many providers spoke about their role in patient education including teaching patients about breast awareness, breast self-examination and the importance of regular Pap smears and allaying fears about the procedure, as such education could facilitate early detection of female cancers.

*It is important for us to diagnose or for quick diagnosis or referral, we are depending on the patient because she’s the one who examined herself, her breast at home. So, we teach them how to see the weight of the two breasts, if they are equal and if she sees that there’s a difference, they are not equal, maybe there’s something that is not normal* (Scenario 1 rural)

*Some patients do not want to do a Pap smear because they have a fear of the unknown that the Pap smear is painful this … but I’m going to do clear health education that the patient may see the importance of doing a Pap smear* (Scenario 1 urban)

## Discussion

This is the first study in SA exploring PHC provider interpretation and management of potential breast and cervical cancer symptoms. In responding to the scenarios, our study showed that PHC providers are likely to take a detailed history and perform a comprehensive examination. Cancer was considered as a potential diagnosis, but providers also reflected on the local context as they worked through a differential diagnosis. The burden of disease in SA includes infectious diseases, maternal and child health and injuries and violence, as well as cancer and other non-communicable diseases [26]. The country has a very high prevalence of HIV and STIs, with a particularly heavy burden of disease among women [27, 28]: in 2019, HIV prevalence among women aged 15–49 years was 25% [27], approximately 1.1. million new cases of STIs are treated annually [28] and sexual violence remains a major problem [29, 30]. Given this disease profile, it is not surprising that infectious diseases and sexual violence remained foremost in PHC providers’ minds.

Local studies have shown that women with breast cancer symptoms may visit PHC facilities several times before referral and diagnosis [12, 13]. A particular challenge facing all PHC providers is the overlap between cancer symptoms and symptoms arising from common local health conditions. Abnormal vaginal bleeding, vaginal discharge and lower abdominal pain, all known symptoms of cervical cancer, can also occur in women with STIs and those who have experienced sexual violence. In addition, STIs may occur concurrently with cervical cancer, further complicating symptom interpretation and diagnosis. There are also infections and other conditions with symptoms similar to breast cancer. For example, symptoms such as skin induration, puckering and ulceration can also occur in women with a breast abscess, and with lactational and non-lactational breast infections. Lipodystrophy, a side-effect of some ART, results in a redistribution of body fat with loss over limbs and gain in various sites including the breast [31]. Symptoms of breast cancer in patients on ART may thus be misattributed to lipodystrophy and could contribute to a delay in timely diagnosis. Our study shows that PHC providers do consider cancer as a potential differential diagnosis but the extent to which symptom overlap contributes to delayed diagnosis of breast and cervical cancer at a primary care level in SA requires further investigation.

Within the SA public health care system, once a PHC nurse suspects that a patient may have cancer, referral for further assessment either to a PHC based doctor or to a secondary level service is required. Appropriate well-functioning referral and feedback systems between primary and secondary level providers are thus critical to timely cancer diagnosis. Well organised feedback systems not only facilitate timely diagnosis but can also serve as a means of continuing education for PHC providers. The challenges of cumbersome appointment, referral and feedback systems have been raised previously [32, 33], and our study underscores the urgent need to comprehensively address this. In our study, very few PHC providers were aware of the national breast and cervical cancer policies, highlighting the importance of having mechanisms to ensure providers are aware of policy developments and how these can support them in clinical decision making and management. Health care providers should also use these policies as a guide to inform and support their clinical decision making in recognising and treating breast and cervical cancer.

The COVID-19 pandemic has resulted in an increased use of online communication within the health sector providing new opportunities for remote clinical assessments and support [34]. In the future, greater use of online communication systems could improve timely diagnosis of symptomatic cancer and address the training needs identified by the PHC providers in our study. However, any online support and training strategy has technological and financial implications and requires stable Internet services.

In the UK, various clinical decision support instruments have been developed to help primary care providers identify patients requiring further investigation for possible cancer [35–37]. These include electronic cancer Risk Assessment Tools (RATs) that are developed using data from UK cohort studies and then validated on a separate patient cohort [35–37] as well as artificial intelligence technology-driven symptom management tools [38]. Primary care providers in the UK reported that the RATs were helpful [11], and there is currently a large pragmatic primary care trial underway, investigating their utility and effectiveness. As SA develops electronic medical records and databases at multiple levels of the health system, development of locally relevant RATs becomes a possibility and could facilitate timely diagnosis of symptomatic cancer. SA breast and cervical cancer RATs will need to make provision for the high prevalence of infectious diseases and sexual violence. Developing of a local symptom management and referral e-Tool could further support PHC providers. Where possible, this e-Tool could link with electronic booking systems at referral, with the aim of streamlining the referral process.

Our study is based in one rural and one urban site in SA and insights gained are likely to be transferrable to other such settings in SA. Further, our study provides an in-depth and nuanced understanding of PHC provider’s appraisal and management of possible breast and cervical cancer symptoms – a strength of qualitative research. We chose to use vignettes in the form of clinical scenarios in our study as it allows for provider views and actions to be explored in a less threatening way thereby avoiding potential social desirability bias. Furthermore, the specificity of vignettes allows for the exploration of contextual influences on judgements [22, 23].

## Conclusion

Clinical assessment at PHC level is complex and influenced by local health issues. Providing context-relevant training and support for PHC providers and improving referral and feedback systems could assist timely diagnosis of women with symptomatic breast and cervical cancer.

## List of abbreviations

ART, Anti-retroviral therapy; HIV, Human immunodeficiency virus; PHC, Primary health care; SA, South Africa; STI, Sexually transmitted infection; RAT, Risk assessment tools; UK, United Kingdom.

## Conflicts of interest

The authors declare that they have no conflicts of interest.

## Funding statement

Research reported in this article was jointly supported by the Cancer Association of South Africa, the University of Cape Town and the SA Medical Research Council with funds received from the SA National Department of Health, GlaxoSmithKline Africa Non-Communicable Disease Open Lab (via a supporting grant Project Number: 023), the UK Medical Research Council (via the Newton Fund). Authors retained control of the final content of the publication. The funders had no role in study design, data collection and analysis, decision to publish or preparation of the manuscript.

## Authors’ contributions

JM, FMW, SES, JH and ADM conceptualised and designed the study. JH oversaw data collection. JH coded the transcripts data. SS sorted and coded the clinical data. JM and FMW reviewed the clinical coding. JM prepared the first draft, incorporated revisions and prepared the final draft. All the authors reviewed drafts and approved the final manuscript.

## Appendix A. Vignettes.

**Vignettes and IDI guide**

Prior to description of scenario and prior to the interview remember:

- Greeting and introduction
- Provide background and information on study
- Complete informed consent before proceeding
- Complete socio-demographic data collection sheet
- Each participant to be presented with one breast and one cervical cancer scenario

Interviews to be audio-recorded.

**Introduction to scenario**

I am interviewing PHC providers about management of certain symptoms experienced by women. I am going to describe different scenarios for you. In each scenario, a female patient is seen by a PHC provider at a clinic. I would like you to help me complete the scenario by describing what the primary care provider in the scenario will ask or do at each stage. There are no correct or incorrect answers.

With your permission, I would like to record our conversation today. The information we discuss today will remain confidential and will not be shared with anyone outside of our immediate research team.

**Once participant has consented to record interview say:** I am going to begin recording the interview now. (**State the participant ID, date, location interviewer name and scenario numbers at the start of the recording)**

### Scenario 1

*A 50-year-old woman with a history of diabetes noticed a lump on her right breast. She comes to this clinic, (insert name of clinic), to have the lump on her right breast checked. At the clinic, she is seen by the Nurse, who must assess and manage the patient.*

**Stage 1 – History**

- After the Nurse introduces herself and makes the woman comfortable, can you tell me what questions would the nurse ask the patient to help with the assessment of this patient?
  For each question provided, interviewer to probe
  - Could you explain to me why it is important for the nurse to ask that question?
  - Could you explain how does it help her to manage the patient?
  Prompt
- Is there any other question/s the nurse is likely to ask the patient? Interviewer to keep prompting until there are no further questions.

**Stage 2 – Clinical examination. We are now going to talk about clinical examinations**

*I am continuing with the scenario. The nurse asks the woman if she can examine her.*

- How would the nurse go about doing the clinical examination?
- Can you tell me what the nurse will be looking out for in the clinical examination?
- For each answer ask
  - Why do you think that is important?
  - How do you think it helps the nurse know what to do?
  Prompt:
- Is there anything else the nurse will look for in the examination? Interviewer to continue prompting participant until there are no further response/things to look out for.

**Stage 3: Investigation and referral**

*The nurse finds out that the patient has had a breast lump for 1 month. On examination, she finds a lump in the right breast and nothing else of significance*.

- Can you tell me, how the nurse, who is based at (insert name of clinic), will manage the patient?
- Probes for each response
  - Why will she do this? i.e. why was that decision made?
- Are there any investigations/tests that the nurse could do or order?
  If yes
  - Could you indicate what test the nurse would order? Why would one do this test?
  - Who would do test?Where will the test be done?
  - How does one order this test?
  - How long does it take for results to be available? – What is the average timeframe for results to become available?How will the results be available? – telephone, electronic, post, return to clinic to get results, etc.

**Stage 4: Referral**

I am going back to the scenario*:*

*The nurse is concerned that the patient might have breast cancer and decides to refer her for further assessment:*

- Can you explain how the referral process works? Including who or where the nurse will refer the patient to. Could you discuss any challenges with referring patients like this? What are the challenges?
- Is there anything that makes it easier to refer patients like this patient? If yes, could you describe what makes it easier to refer patients like this patient?Will the nurse get to know the outcome of the referral? If no, why not and if yes, how do you think the nurse will get to know the outcome of the referral?
- Is there anything else you want to add to this scenario? Could you discuss

We have come to the end of this scenario.

### Scenario 2

*A 50-year-old woman with a history of diabetes is worried about changes on the skin of her right breast. She comes to this clinic, (insert name of clinic), to have this checked. At the clinic she is seen by a nurse, who must assess and manage her.*

**Stage 1 – History**

- After the nurse introduces herself and makes the patient comfortable, can you tell me what questions she will ask the patient to help with her assessment?

For each question provided, interviewer to probe

- Why is it important for the nurse to ask that question?
- How does it help her to manage the patient?

Prompt

- Is there any other question the nurse is likely to ask the patient? Interviewer to probe until there are no further questions.

**Stage 2 – Clinical examination**

*I am continuing with the scenario. The nurse asks the woman if she can examine her.*

- How would the nurse go about doing the clinical examination?
- What could the nurse be looking out for in the clinical examination?

For each answer ask?

- Why is that important?
- How does it help the nurse to know what to do?

Prompt:

- Is there anything else that the nurse could look for in the examination? Interviewer to keep asking this until there are no further response/things to look out for.

**Stage 3: Investigation and referral**

*The nurse finds out that the patient had noticed the skin changes on her right breast 1 month ago. On examination, the nurse notices the following change in skin on right breast (show picture of breast skin orange peel appearance). She finds nothing else of significance*.

- How will the nurse, who is based at (insert name of clinic), manage the patient?

Probe – for each response

- Why will she do this? i.e. why was that decision made?

- Are there any investigations/tests that the nurse could do or order?

If yes

- What test?
- Who would do test?
- Where will the test be done?
- How does one order this test?
- When will the result be available?
- How will the results be available? – telephone, electronic, post, return to clinic, etc.

**Stage 4: Referral**

*In this scenario, the nurse decides to refer the patient for further assessment:*

- Can you describe how this referral process works?Who will she refer to?
- Any challenges with referring patients like this? What are the challenges?
- Is there anything that makes it easier to refer patients like this patient?Is the nurse likely to get feedback from the referral centre?
- Will the nurse get to know the outcome of the referral?
- Is there an appointment system for making referrals?
- Is there anything else you want to add to this scenario?

### Scenario 3

*A 35-year-old woman who is HIV positive comes to the clinic (insert name of clinic) because she has had a yellow vaginal discharge and vaginal bleeding after sexual intercourse for the past month. The woman is seen by the nurse.*

**Stage 1 – History**

- After the nurse introduces herself and makes the patient comfortable, what questions is she likely to ask the woman to help with the assessment?

For each question provided, interviewer to probe

- Why do you think it is important for her to ask that question?
- How does it help her to manage the patient?

Prompt

- Is there any other question that the nurse is likely to ask the patient? Interviewer to continue probing until there are no further questions.

**Stage 2 – Clinical examination**

*I am continuing with the scenario. The nurse asks the patient if she can examine her.*

- How would the nurse go about doing a clinical examination?
- What could she be looking out for in the clinical examination?

For each answer ask?

- Why do you think this is important?
- How does it help her know what to do?

Prompt:

- Is there anything else that the nurse will look for in the examination? Interviewer to keep asking this until there are no further response/things to look out for.

**Stage 3: Investigation**

*The nurse does a speculum examination and notices a large lesion blocking her view of the cervix. There is blood oozing from the lesion.*

- Could you describe how the nurse, who is based at (insert name of clinic), would manage the patient?

Probe

- Why will she do this? i.e. why was that decision made?
- Are there any investigations/tests that she could do or order?

If yes

- What kinds of tests might be ordered?
- Who will do the test?Where will the test be done?
- When would the result be available?
- How will it be available? Telephone, electronic, post, etc.

**Stage 4: Referral**

*I am continuing with the scenario. The nurse is concerned that the patient might have cervical cancer and decides to refer her for further management.*

- Could you describe how the referral process works?
- Is there some sort of appointment system?
- Who will the nurse refer the patient to?
- How far is the referral centre?
- Are there any challenges with referring patients like this?

If yes

- What are the challenges?
- Is there anything that makes it easier to refer patients like this patient? If yes, could you describe what these might be?

- Is the nurse likely to get feedback from the referral centre? If yes, how long would this process take?
- Will the nurse get to know the outcome of the referral?
- Is there anything else you want to add to this scenario?

### Scenario 4

*A 60-year-old woman comes to the clinic (insert name of clinic) because she has vaginal bleeding. She is worried because her periods stopped more than 10 years ago so she cannot understand why she has started bleeding again. The patient is seen by the Nurse.*

**Stage 1 – History**

- After the nurse introduces herself and makes the patient comfortable, what questions will she ask to help with the assessment?

For each question provided, interviewer to probe

- Why is it important for her to ask that question?
- How does it help her to manage the patient?

Prompt

- Is there any other question the nurse is likely to ask the patient? Interviewer to continue probing this until there are no further questions.

**Stage 2 – Clinical examination**

*I am continuing with the scenario. The nurse asks the patient if she can examine her.*

- How would the nurse go about doing the clinical examination?
- What will the nurse be looking out for in the clinical examination?

For each answer ask?

- Why is that important?
- How does it help her know what to do?

Prompt:

- Is there anything else the nurse will look for in the examination? Interviewer to keep asking this until there are no further response/things to look out for.

**Stage 3: Investigation**

*The nurse cannot see anything of significance on clinical examination.*

- How will the nurse, who is based at (insert name of clinic), manage the patient?

Probe

- Why will she do this? i.e. why was that decision made?
- Are there any investigation/tests that she would do or order?
- If yes What test?
  - Who will do the test?
  - Where will the test be done?
  - When would the result be available?
  - How will it be available? Telephone, electronic, post, etc.

**Stage 4: Referral**

*Coming back to the scenario. The nurse does a Pap smear and the result comes back as invasive carcinoma of the cervix.*

- How will the nurse inform the patient of the result?
- How will the patient be managed?
- Where will she be referred to for further management?
- How does the referral process work?
- Any there any challenges with referring patients like this?If Yes – What are the challenges?
  - Is there anything that makes it easier to refer patients like this patient?Is the nurse likely to get feedback from the referral centre?
- Will the nurse get to know the outcome of the referral?
- Is there anything else you want to add to this scenario?

**Post-scenario questions**

**The following questions are to be asked at the end of the interview after the participant has completed two scenarios**

1. Are you aware of any policies or guidelines for the management of patients with suspected breast cancer in SA?

If Yes

- Could you tell me the name of the policy/guideline? And date of guideline?
- Can you briefly describe what the policy/guideline says?

2. Are you aware of any policies or guidelines for the management of patients with suspected cervical cancer in SA?

If Yes

- Could you tell me the name of the policy/guideline? Could you tell me the date of the guideline?
- Can you briefly describe what the policy/guideline says?

Thank participant and close interview.

## Appendix B. Matrix framework

**Table:**

Participant number	Scenario	History	Examination	Investigations
P001	1	Duration of lump, any progression in size, bleeding, itchiness or discharge? Family history of cancer	Breast examination: is it painful? Mobile? Tender? Signs of inflammation? Pus? Bleeding? Location? Skin changes in the area.	Blood sugar. Blood pressure and vitals.
P004	1	Duration of lump, any pain in the lump? Menstrual history- still having periods or not? Family history of cancer?	Breast examination: feel lumps and characterise it. Check for any discharge/blood, asymmetry, dimples on the breast.	Blood sugar. Vitals.
P005	1	When did the lump start? how long has it been there? Painful? Irritating or itchy? Is there discharge? Does the pain change during the night/day? Characterise the pain: how long does the pain last? Is it transient pain? Is the patient on any medications? (Antibiotics/painkillers/ointments) Any other symptoms: Dizziness, fatigue, tiredness?	Breast examination of both breasts and both armpits by nurse, then referral to clinic gynae doctor (may re-examine).	Mammogram and ultrasound referral by doctor at the clinic. Bloods including Hb and platelet count. Follow up within 1 week of results.
P007	1	Duration of lump, hard/soft lump? Any tenderness? Swelling? Ask TB and HIV status Family history of cancer Any previous Pap smears and their results	Breast examination: palpate the lump, skin examination, any enlargement of the breast, any pain? size? Palpate the armpits. Any discharge, soft/hard? Any swelling? Check nipples for any bleeding/discharge. Any scarring. Rash/scaly skin?	TB and HIV test
P009	1	Pain in the lump? Duration of lump. Family history of cancer. Sexually active? Pre or post-menopause?	Full physical examination from “head to toe”: palpate neck, lymph nodes, look for signs of anaemia, cyanosis, jaundice, look in the mouth, check for leg oedema Breast examination: palpate both breasts	Pap smear to check for cervical cancer. Check adherence to diabetic treatment.
P010	1	When did you first notice the lump? Progression in size? Any pain? Past medical history. Family history of cancer. Any other lumps? Any abnormal bleeding? Lower abdo pains?	Breast examination of both breasts: palpation of lump, consistency, mobile? ‘Check whether it is an abscess’	No clinic investigations.
P012	1	Is it painful? Is it hard/soft? Any bruises/open wounds/ulcers? Any pus/discahrge? Pre or post-menopausal? Sexually active? Family history of cancer	Breast examination: Palpate the lump, check for signs of inflammation: hot? Red? Check colour of the breast (red for inflammation, blue for cyanosis). Check armpits.	Random blood sugar. Urine dipstick. Urine for pregnancy. HIV test.
P015	1	When did the lump develop? Painful? Unusual secretions? Asymmetry? Still menstruating or breastfeeding? Are you currently pregnant? Family history of cancer.	Breast examination: asymmetry? Scars? Abnormal rashes? Palpate the lump, nipple discharge? Full physical examination and systems evaluation: anaemia, eczema, clubbing,	Vital signs, urine dipstick, blood test for HIV, and glucose. May do cervical screening if you suspect at risk for cancer
P017	1	Any pain? Are you breastfeeding? Any previous episodes? Any previous treatments for the lump (e.g. any traditional medicines? Any other doctors visited?). Any secretions? Any swelling?	Breast examination: is it swollen? Red? Any sores? Discharge? Distended veins? Palpate for any tenderness? Lump: fixed/mobile? Tender? Size of lump? Obstetric history: how many children? How many alive? Method of delivery? Gynaecology history: menstrual cycle history, lower abdo pains, abnormal vaginal discharge? If there is discharge, characterise it and refer for papsmear.	Vital signs. Blood sugar. HIV test. TB test.
P019	1	Any previous breast lumps? Any pain? Any secretions from the breast? Colour and size of the lump? Is the lump rigid? Is the pt breastfeeding? Any allergies? Any recent history of insect bites to the breast area? Family history of cancer or any breast conditions? Any previous surgeries esp breast surgery? Any systemic symptoms: Urinary? Respiratory? Lifestyle questions: Alcohol use? Ideas and concerns of the patient, any related stress/depression	Breast examination of both breasts. General whole body examination. Lymph node examination.	HIV test. Blood sugar. Bloods: Haemoglobin Cervical screening if appropriate.
P020	1	When did it start? Pain? Any swelling? Is it growing? Is it mobile? Family history of cancer? Does she smoke?	Breast examination: size, shape, swelling? Redness? Rash especially rash like an insect bite? Any scaling? Nipple discharge? Bloodly discharge? Inverted nipples? Examine the armpits	Vital signs
P022	1	Any pain? If yes, for how long. Any changes in the breast: breast tenderness, any change to the nipples, any discharge? Is the lump mobile? How long has it been present? Family history of breast cancer	Breast examination of both breasts and armpits: any pain? discharge? Asymmetry? Abnormalities in the shape of the breasts? Colour change in breasts?	None by nurse.
P023	1	Is she a smoker? Any medications including herbal/traditional? Family history of cancer (breast, ovarian or prostate especially)? Gynae history: Last menstural period? Any use of contraception- if so what form? Obs history: How many pregnancies? Was she breastfeeding? Any problems during breastfeeding?	Breast examination: look around the armpits; examine breast and skin, lymph nodes. Check for any surgical scars. Any nipple discharge? Pain on palpation?	None by nurse.
P002	2	When did she notice the skin changes? Is it getting better or worse? Other symptoms: pain? Change in size of breast? Any discharge? Any breast lumps? Menstrual history: last menstrual period, pre or post menopausal? Last pap smear? Obs: Did she breastfeed her kids? Family history of cancer?	Breast examination of both breasts and armpits: check for any enlarged lymph nodes, discharge, lumps? Check the colour of the breast and areola and discharge	Cervical exam. HIV test. If there is breast discharge, send a smear. Bloods for Hba1c if not done in the last 6 months.
P003	2	Any changes in the nipple or the skin? Any breast lumps? Any enlarged nodes? Is the patient pregnant? Family history of breast cancer. Medication history (especially certain antihypertensives and oral contraceptives which can cause nipple discharge)	Breast examination: any skin changes? Breast lumps? If yes, is it fixed? Irregular? Lymph nodes in the armpits? Any changes to the nipple? Any sores on the skin? Any discharge?	No investigations
P006	2	Diabetes history: medication, type of diabetes, how long has she been on treatment, family history of diabetes? Obs history: how many children? Method of delivery? Did she breastfeed? How long did she breastfeed for? When did she start being sexually active? Any surgeries? Any history of cancer? Pap-smears: are they up to date? what are the results? Family history of cancer? Menstrual history: time of menarche? Post- or pre menopausal?	Breast examination of both breasts and armpits: asymmetry? Any nipple changes? Any discharge from nipples? Any breast lumps? Any skin discolourations? Any signs of inflammation? Female internal examination: look for any discharge? Warts?	Vital signs. Blood sugar levels. Cervical smear. Doctor will take bloods to check for inflammation and request a mid-stream urine test . Bloods can be fast-tracked.
P008	2	Family history of breast cancer? Any previous breast lumps/cysts? When did she last do self-breast examinations? Has she ever used/still using any form of contraception? If yes, what type? Obs history: how many pregnancies? How many live births? Smoking and alcohol use? Diabetes control: when did she check her sugar? Is there adequate control on her current medications?	Breast examination of both breasts: size of any lumps? Colour of breast?	Vital signs. Urine test. Finger prick glucose.
P011	2	How long ago did she notice the change? Any pain? Any difference between the breasts? Any previous incidences of breast changes? Family history of cancer? Diabetes history: how is the control? taking medications? is the treatment helping? Explore patient ideas/any misconceptions/fears about cancer and its implications.	Breast examination of both breasts: any pain/tenderness? any lumps? Look at the skin changes: colour, peeling, rashes? How is it different to the other breast? Any discharge? Is the secretion from the whole or part of the nipple? Check lymph nodes. Full physical examination: look at mouth, ears, eyes.	Cervical cancer screening. HIV test. Vital signs including blood pressure check.
P013	2	How long has the skin been like this? When did it start to change? Was she bitten? Is she on anti-retroviral treatment? Any sores/wounds? Ask about food and nutrition (for diabetes management). How is her personal hygiene? How is she managing at home? Is she breastfeeding?	Breast examination: is there a lump? How big is it? Check the skin change. Check the whole body for other skin changes.	HIV test.
P014	2	Duration of skin change? Is there pain- if so, characterise the pain (type of pain, location of pain). Any medications. Any previous episodes of breast skin changes? Is she breastfeeding? Any discharge/secretions? Diabetes history: type? How is the control? What treatment are they on? Is it manageable? Adherence to treatment?	Breast examination: any asymmetry (size and shape of the breast)? Any signs of inflammation (swelling, redness, pus)? Any lumps? Any secretions?	No investigations
P016	2	Duration of skin change? Is it painful? Is it itchy or any signs of infection? Is she breastfeeding? Is she sexually active? Do they use condoms?	Breast examination: lumps on the breast? Is the rash from inside the breast or not? Full examination: head, eyes, mouth (check for anaemia/other problems)	HIV test.
P018	2	Ask her what she felt. Family history of cancer. Is it painful? Is it mobile? Is there discharge from the nipples? Are there any lumps? Any past medical history? Any previous breast issues or cracked nipples? Are you breastfeeding?	Breast examination: examine the skin, signs of swelling, nipple discharge, any tenderness	Vital signs. If discharge, swab the discharge.
P021	2	What are the breast changes? Painful? Duration of changes? Does she know how to do self-breast examination? Post or pre-menopausal. Any previous pap smears. HIV status. Any forms of contraception used? Is she breastfeeding? Has she had her screening mammogram?	Breast examination: any scars, any lumps, any discharge? Signs of inflammation (warm to touch, swelling, tenderness)?	Pap smear
P024	2	Any pain in her breast? Does she examine her breasts regularly? Are any any lumps? What is the change in colour? Any change in her treatment (i.e. any use of new medications)? Family history of cancer?	Breast examination of both breasts and the armpits: any lumps? Any change in texture of the skin?	No investigations
P001	3	Any unprotected sexual intercourse? Where is the discharge coming from? Duration of discharge? What colour? Duration of bleeding? Any abdominal pain? Up to date on pap smear? Any trauma to the uterus or abdomen? Contraception: how long has it been used for? What form of contraception? Is she pregnant?	Full physical examination including breast examination Abdominal tenderness Bimanual examination Speculum examination to look at cervix. Discharge: colour? Where is it from? Any changes to the cervix?	Vitals including blood pressure check Bloods: heamoglobin RPR for syphilis, urine pregnancy test Pap smear or swab discharge
P004	3	Is she using condoms? Has she been councelled regarding positive HIV status? How many partners? Are partners aware of HIV+ status? Is she on antiretroviral therapy (ARVS)?	Vaginal examination to see the discharge: colour, check for any sores, check for any obvious abnormalities, any pubic lice? Any warts?	Urine dipstick , syphilis test RPR
P005	3	When did it start? How long for? Is intercourse painful? Does she use protection during sex? Has this happened before? Has she been to clinic for this problem before? If so, any previous treatments? Any previous Pap smears? If so, what was the result? How many partners?	Abdominal palpation for tenderness Speculum examination: any redness, pus? Bleeding? How much bleeding? Vaginal examination: check for lumps, sores, oozing, blisters	Pap smear, bloods such as Hb, vital signs. Speculum examination: ask the doctor to do pipelle biopsy and swab pus
P006	3	Gynae history: when did she start being sexually active? When was menarche? HIV: When did she find out she was positive? Is she on treatment? When did she start treatment? Is she using condoms? What is her partner’s HIV status? How many sexual partners? Have she been non-compliant in the past? Discharge: when did it start? What did she notice? What colour? Does it smell? Has she had treatment for this before? Last pap smear?	Speculum examination Bimanual examination: palpate for any lumps, observe colour and smell of discharge, any warts? Breast examination	Pap smear. Get gynae doctor to do colposcopy and perform biopsy in clinic.
P008	3	HIV: when diagnosed? On treatment? Where is she taking the treatment? How many sexual partners? Is she using condoms? When was the last CD4 count and viral load blood test? Any pap smears done? Discharge: when did she notice it? Any other symptoms? Any abdominal pain? Itchiness? Other problems? Bleeding: when did it start? Any use of contraception. If yes, for how long.	Speculum examination: where is the discharge and bleeding from? How does it look? Abdominal examination: any pain/tenderness? General appearance of patient	Bloods: creatinine levels, viral load, CD4 count Pap smear after the bleeding has stopped but not now when the patient is bleeding.
P010	3	HIV: is she taking treatment? Is she using a condom? How many sexual partners? Bleeding: When did it start? Any previous pap smears? How is her personal hygiene?	Physical examination of the whole body: Pale? Sores on the body? Rash Vaginal examination: any vaginal warts? Sores? Signs of fungal infection?	Pap smear Blood test for syphilis
P012	3	Duration of symptoms? When did it start? Any lower abdo pain? Any pain when urinating? Is she using condoms? Last pap smear? Sexual history: is she using condoms? Family history of cancer? Obs: how many children? Are you pregnant? Method of delivery? Any prior radiation (needed for pap smear form)?	Vaginal examination: any warts? Ulcers? Discharge?	Pregnancy test Pap smear- if pap smear unsuccessful due to blood obscuring the cervix, refer to doctor at the nearest hospital. Random blood sugar. Bloods: Hemoglobin
P014	3	When did she last have sex? Did she use a condom? How is the vaginal discharge? Any odour? Any lower abdominal pain? Any burning during menstruation? Any itching? HIV: on treatment? Adherent? Married? Is the partner HIV positive? If so, is he on treatment? Is she using condoms? How many partners? Menstrual cycle: how long?	Vaginal examination: any warts? Signs of inflammation? Amount of discharge? Consistency of discharge? Odour? Any redness?	Pap smear Screen for TB
P016	3	Any previous Pap smears- if so, when was last one? Are the partners having a similar problem	Vaginal examinaion: is it yellow? Sticky? Odour? Bleeding? How much bleeding? Speculum examination: pap smear	Bloods: viral load
P017	3	Last menstrual period? Any contraceptives? Duration of symptoms? Is this the first time she is getting treatment for this? Any itching/burning? HIV: on anti-retrovirals? Latest viral load?	Vaginal examination: any discharge? Sores? Odour to the discharge? Colour of the blood? Signs of STI?	Papsmear Urine pregnancy test Bloods: viral load, RPR for syphilis, Hemoglobin
P019	3	Pain or burning when passing urine or during menstruation? Any lesions/lumps around the vagina? Any vaginal warts?Any obvious asymmetry to the labia?Any lower abdo tenderness? Any visible haematuria? Does post-coital bleeding happen regardless of lubrication/foreplay? Any history of trauma to the pelvis? Date of last menstrual period? Any other pain? Discharge: itchy? malodour? Any history of thrush? Family history of cervical cancer? Past medical history esp of cancer? Any previous Pap smears?	Vaginal examination: discharge? Malodour? Check any asymmetry in the labia, palpate to ensure no lumps	Urine tested for urinary tract infection (dipstick/mid-stream urine) Pregnancy test Bloods: hb
P021	3	HIV: when diagnosed? On treatment? Is viral load suppressed? When was the last pap smear? Was it normal? Has she disclosed HIV status to her partner? HIV status of partner? If positive, is he on anti-retroviral treatment? Is she using condoms regularly? Any use of contraception? Any signs of TB?	Vaginal examination: examine the discharge, any sores/genital warts? Any abnormalities around the vulva?	TB screening, Pap smear, RPR for syphilis
P023	3	When was she last sexually active? Is she using condoms? Other contraceptives? Is the discharge smelly? HIV status of partner?	Vaginal examination: any sores/warts? Bimanual examination: check discharge to see colour and odour? Any cervical tenderness? Lower abdominal pain?	Pregnancy test, RPR for syphilis. Urine test.
P002	4	Was she on any contraceptives? Is she sexually active? Pain with the bleeding? Clotted blood? Headache? Dizziness? Blurred vision? Colour of blood: bright red, dark red? How many pads is she changing a day? Number of sexual partners? Any chronic diseases?	Head to toe physical examination- signs of anaemia and cyanosis, any difficulty breathing? Skin colour? Pallor? Check eyes for anaemia. Check dryness of mouth? Any abdominal distention/tenderness? Any abdominal guarding? Vaginal examination: is the os closed or open? Speculum examination and bimanual examination. Check for uterine prolapse. If nothing of note on examination, get doctor to double check.	Vital signs. Pap smear. Bloods: Full blood count especially haemoglobin and platelets. Check HIV status (mentioned after in scenario patient was said to have invasive carcinoma)
P003	4	Last Pap smear? What was the result? Family history of cervical cancer?	Speculum examination: check the cervix for an erosion/tenderness? Any abnormal discharge? Any genital warts?	Pap smear
P007	4	How long have you been bleeding for? How much blood is there? Any triggers? It is painful? Are you sexually active? Family history of cervical cancer?	Speculum examination: does the cervix look healthy? Is there bleeding? How much? Any vaginal warts?	Pap smear after giving the patient oral progesterone to stop the bleeding. HIV positive. HIV test. (If positive, do CD4 count and creatinine, and start anti-retrovirals)
P009	4	When did the bleeding start? Is it continuous or episodic? Is she on any medications (e.g. antiretroviral treatment? Diabetic meds? Hypertension meds?)?	None suggested by nurse initially. When prompted, head to toe examination: check for cyanosis, jaundice, palpate the breasts, palpate lymph nodes	None
P011	4	Any history of assault/rape? Duration of bleeding? Severity? Heavy flow? Regular flow/spotting? Any clots? Any previous pap smears? What were the results? Any past medical history (e.g. diabetes, hypertension, HIV and AIDS)? Headaches? Dizziness? vomiting?	Examine fully: check for anaemia. Weigh the patient to check for unexpected weight loss. Speculum examination: any spots/sores/unusual redness? Clots of blood? Vaginal itching? Genital warts/sores?	Pap smear
P013	4	When was the last pap smear? Is she sexually active? How many partners?	Speculum examination Breast examination	Pap smear
P015	4	When did the bleeding start? Family history of cancer? Any previous pap smears- if so, what was the result? Any pain? Any abdominal pain? Obstetric history: how many children? Method of delivery? How were your periods when you were menstruating? Any use of vaginal tobacco?	Phsyical examination: signs of anaemia If pad used: check for soaking, when was last changed? Speculum examination: cervix colour, shape, any abnormalities, any pain?	Urgent pap smear- results back in 2 weeks if you phone the centre and ask for it to be processed urgently. Bloods: Haemoglobin.
P018	4	Any rape/assault/trauma? Any pain? Any sores? If so when did it start? Any thrush? Any vulval itching?	Speculum examination: any sores?	Pap smear. If there is itching, do the glucose test for diabetes.
P020	4	When did the bleeding start? Family history of cancer? Smoker? Is this the result of rape? Any scratching to the vulval region?	Nurse did not want to answer	Pap smear.
P022	4	How long is the bleeding? Any previous Pap smears? Family history of cancer? Any contraceptives?	Speculum examination	Vitals. Pap smear. Bloods: Haemoglobin
P024	4	Any rape/trauma? Duration of bleeding? When did it start? Any pain? What soap does she use when she washes?	Male nurse would refer to a female nurse to examine.	Pap smear by female nurse/

## References

[1] Bray F, Ferlay J, Soerjomataram I (2018). Global cancer statistics 2018: GLOBOCAN estimates of incidence and mortality worldwide for 36 cancers in 185 countries. CA Cancer J Clin.

[2] South African National Cancer Registry (2017). Cancer in South Africa 2017. https://www.nicd.ac.za/centres/national-cancer-registry/.

[3] South African National Department of Health, South Africa (2017). Breast cancer: prevention and control Policy.

[4] South African National Department of Health (2017). Cervical cancer prevention and control policy.

[5] World Health Organization (WHO) (2020). WHO report on cancer: setting priorities, investing wisely and providing care for all.

[6] Mash R, Ogunbanjo G, Naidoo SS (2015). The contribution of family physicians to district health services : a national position paper for South Africa : forum. S Afr Fam Pract.

[7] Koo MM, Hamilton W, Walter FM (2018). Symptom signatures and diagnostic timeliness in cancer patients: a review of current evidence. Neoplasia.

[8] Koo MM, Unger-Saldaña K, Mwaka AD (2021). Conceptual framework to guide early diagnosis programs for symptomatic cancer as part of global cancer control. J Glob Oncol.

[9] Rubin G, Berendsen A, Crawford SM (2015). The expanding role of primary care in cancer control. Lancet Oncol.

[10] Nekhlyudov L, Latosinsky S (2010). The interface of primary and oncology specialty care: from symptoms to diagnosis. J Natl Cancer Inst Monogr.

[11] Hamilton W (2010). Cancer diagnosis in primary care. Br J Gen Pract.

[12] Moodley J, Cairncross L, Naiker T (2016). Understanding pathways to breast cancer diagnosis among women in the Western Cape Province, South Africa: a qualitative study. BMJ Open.

[13] Moodley J, Cairncross L, Naiker T (2018). From symptom discovery to treatment - women's pathways to breast cancer care: a cross-sectional study. BMC Cancer.

[14] Pruitt LCC, Odedina S, Anetor I (2020). Breast cancer knowledge assessment of health workers in Ibadan, Southwest Nigeria. JCO Glob Oncol.

[15] Onyenwenyi AOC, McHunu GG (2019). Primary health care workers' understanding and skills related to cervical cancer prevention in Sango PHC centre in south-western Nigeria: a qualitative study. Prim Health Care Res Dev.

[16] Martin AN, Kaneza KM, Kulkarni A (2019). Cancer control at the district hospital level in Sub-Saharan Africa: an educational and resource needs assessment of general practitioners. J Glob Oncol.

[17] Moodley J, Constant D, Mwaka AD (2021). Anticipated help seeking behaviour and barriers to seeking care for possible breast and cervical cancer symptoms in Uganda and South Africa. Ecancermedicalscience.

[18] Harries J, Scott SE, Walter FM (2020). Women's appraisal, interpretation and help-seeking for possible symptoms of breast and cervical cancer in South Africa: a qualitative study. BMC Women's Health.

[19] Mwaka AD, Walter FM, Scott S (2021). Symptom appraisal, help-seeking and perceived barriers to healthcare seeking in Uganda: an exploratory study among women with potential symptoms of breast and cervical cancer. BMJ Open.

[20] Moodley J, Constant D, Mwaka AD (2020). Mapping awareness of breast and cervical cancer risk factors, symptoms and lay beliefs in Uganda and South Africa. PLoS One.

[21] Moodley J, Scott SE, Mwaka AD (2019). Development and validation of the African Women Awareness of CANcer (AWACAN) tool for breast and cervical cancer. PLoS One.

[22] Finch J (1987). The vignette technique in survey research. Sociology.

[23] Hughes R, Huby M (2012). The construction and interpretation of vignettes in social research. Soc Work Soc Sci Rev.

[24] Gale NK, Heath G, Cameron E (2013). Using the framework method for the analysis of qualitative data in multi-disciplinary health research. BMC Med Res Methodol.

[25] Tong A, Sainsbury P, Craig J (2007). Consolidated criteria for reporting qualitative research (COREQ): a 32-item checklist for interviews and focus groups. Int J Qual Health Care.

[26] Pillay-van Wyk V, Msemburi W, Laubscher R (2016). Mortality trends and differentials in South Africa from 1997 to 2012: second national burden of disease study. Lancet Glob Health.

[27] UNAIDS Country factsheet. https://www.unaids.org/en/regionscountries/countries/southafrica.

[28] Let our actions count South Africa's national strategic plan for HIV, TB and STIs: 2017–2022. https://sanac.org.za//wp-content/uploads/2017/06/NSP_FullDocument_FINAL.pdf.

[29] Steele SJ, Abrahams N, Duncan K (2019). The epidemiology of rape and sexual violence in the platinum mining district of Rustenburg, South Africa: prevalence, and factors associated with sexual violence. PLoS One.

[30] Dunkle KL, Jewkes RK, Brown HC (2004). Gender-based violence, relationship power, and risk of HIV infection in women attending antenatal clinics in South Africa. Lancet.

[31] Zinn RJ, Serrurier C, Takuva S (2013). HIV-associated lipodystrophy in South Africa: the impact on the patient and the impact on the plastic surgeon. J Plast Reconstr Aesthet Surg.

[32] Moodley J, Constant D, Botha MH (2019). Exploring the feasibility of using mobile phones to improve the management of clients with cervical cancer precursor lesions. BMC Women's Health.

[33] Momberg M, Botha MH, Van der Merwe FH (2017). Women's experiences with cervical cancer screening in a colposcopy referral clinic in Cape Town, South Africa: a qualitative analysis. BMJ Open.

[34] Nnaji CA, Moodley J (2021). Impact of the COVID-19 pandemic on cancer diagnosis, treatment and research in African health systems: a review of current evidence and contextual perspectives. Ecancermedicalscience.

[35] Hamilton W, Green T, Martins T (2013). Evaluation of risk assessment tools for suspected cancer in general practice: a cohort study. Br J Gen Pract.

[36] Hippisley-Cox J, Coupland C (2013). Symptoms and risk factors to identify women with suspected cancer in primary care: derivation and validation of an algorithm. Br J Gen Pract.

[37] Hippisley-Cox J, Coupland C (2015). Development and validation of risk prediction algorithms to estimate future risk of common cancers in men and women: prospective cohort study. BMJ Open.

[38] https://cthesigns.co.uk.

